# The Potential Therapeutic Value of Aspirin in Anaplastic Thyroid Cancer

**DOI:** 10.3390/cancers16244203

**Published:** 2024-12-17

**Authors:** Enke Baldini, Silvia Cardarelli, Eleonora Lori, Elena Bonati, Federica Gagliardi, Daniele Pironi, Poupak Fallahi, Alessandro Antonelli, Vito D’Andrea, Salvatore Ulisse, Salvatore Sorrenti

**Affiliations:** 1 Department of Surgery, “Sapienza” University of Rome, 00161 Rome, Italy; enke.baldini@uniroma1.it (E.B.); silvia.cardarelli@uniroma1.it (S.C.); eleonora.lori@uniroma1.it (E.L.); federica.gagliardi@uniroma1.it (F.G.); daniele.pironi@uniroma1.it (D.P.); vito.dandrea@uniroma1.it (V.D.); salvatore.sorrenti@uniroma1.it (S.S.); 2 General Surgery Unit, Department of Medicine and Surgery, Parma University Hospital, 43126 Parma, Italy; ebonati86@gmail.com; 3 Department of Translational Research and New Technologies in Medicine and Surgery, University of Pisa, 56126 Pisa, Italy; poupak.fallahi@unipi.it; 4 Department of Surgery, Medical and Molecular Pathology and Critical Area, University of Pisa, 56126 Pisa, Italy; alessandro.antonelli@unipi.it

**Keywords:** anaplastic thyroid cancer, aspirin, therapy

## Abstract

**Background:** several experimental findings and epidemiological observations indicated that aspirin/acetylsalicylic acid (ASA) may be endowed with anticancer effects against a variety of human malignancies, including thyroid carcinomas. Among these, undifferentiated/anaplastic thyroid carcinoma (ATC) is one of the most aggressive and lethal human cancers, refractory to all currently available therapies. **Methods:** we here evaluated in a preclinical setting the effects of ASA on a panel of three ATC-derived cell lines: the CAL-62, the 8305C, and the 8505C. **Results:** the data obtained demonstrated the ability of ASA to inhibit, in a dose- and time-dependent manner, the proliferation of all ATC cell lines investigated, with IC_50_ values comprised between 2.0 and 4.3 mM. Cell growth was restrained with the same efficacy when the ASA treatment was applied to three-dimensional soft-agar cultures. In addition, ASA significantly reduced migration and invasion in two of the three ATC cell lines. We finally investigated the effects of ASA on the MAPK and PI3K/Akt signaling pathways, which are often altered in ATC. The results showed that the phosphorylation status of the Akt1/2/3 kinases was significantly reduced following ASA treatment, while ERK1/2 phosphorylation was either unaffected or slightly upregulated. **Conclusions:** our findings support epidemiological evidence on the anticancer potential of ASA. On this basis, further investigations should be carried out to assess the usefulness of ASA as adjuvant therapy in patients affected by ATC.

## Simple Summary

Anaplastic thyroid cancer (ATC) is among the most aggressive human cancers, with a very poor prognosis. Over the last decades, several experimental and clinical findings have been accumulated supporting the therapeutic role of aspirin/acetylsalicylic acid (ASA) against different human neoplasms, including thyroid cancer. Here, we show that treatment with ASA inhibits the proliferation and survival of a panel of three ATC-derived cell lines cultured in vitro. It can also hamper cell migration and invasion in two of the three cell lines and strongly impair their capability of forming 3D masses within a solid milieu. Moreover, we demonstrated that ASA reduces the intracellular activity of a biochemical pathway known to be involved in ATC cell replication. In conclusion, the findings reported here warrant further investigations to fully evaluate the potential therapeutic value of aspirin in ATC patients.

## 1. Introduction

Acetylsalicylic acid (ASA), launched on the market by Bayer in 1899 with the trademark “aspirin”, is still one of the most commonly used drugs in the world due to its analgesic, antipyretic, anti-inflammatory, and antithrombotic properties [1,2]. The latter is known to be mediated by irreversible aspirin-induced acetylation of cyclo-oxigenase-1 (COX-1) and cyclo-oxigenase-2 (COX-2), which leads to the inhibition of prostaglandin and thromboxane production [1,2]. Besides this canonical mechanism of action, ASA has also been shown to affect inflammatory/immunological processes and possess antiviral effects against RNA and DNA viruses, mainly through inhibition of the nuclear factor-kB (NF-kB) signaling pathway [2].

Several experimental and clinical findings indicate that ASA may also be endowed with anticancer properties. The first evidence came from a study by Gasic and colleagues, who demonstrated the ability of ASA to significantly reduce the number of metastases in tumor-bearing mice [3,4]. These authors hypothesized that such an effect was associated with the inhibition of platelet formation by ASA [4]. Later on, aspirin and other nonsteroidal anti-inflammatory drugs capable of inhibiting COX-2 were found to be effective in reducing tumor growth and metastases in animal models bearing human colon cancer xenografts [5,6]. The first epidemiological association between aspirin-containing medications and cancer emerged in 1988 from a study by Kune and colleagues, who analyzed the relationship between colorectal cancer (CRC) and various drugs [7]. They reported that the use of aspirin-containing medications was significantly less prevalent in CRC patients than in age- and sex-matched controls [7]. Over the following years, the anticancer potential of aspirin was further explored by several studies, some of which have produced encouraging evidence and others have not [8].

In 2016, Elwood and colleagues performed a meta-analysis of five randomized clinical trials and forty-two observational studies on aspirin in different cancer types, including CRC, breast, and prostate cancers [9]. This study supported the efficacy of aspirin in significantly reducing the cause-specific mortality from colon, breast, and prostate carcinomas. These findings were corroborated by a second meta-analysis reported in 2021 by the same authors, including 118 observational studies on 18 different cancer types [10]. In fact, cancer patients taking aspirin displayed a 20% reduction in mortality, and this effect was not limited to one or a few cancer types, suggesting that aspirin should deserve consideration as adjuvant therapy in cancer treatment [11].

Epithelial thyroid cancer is the most frequent endocrine malignancy, with an incidence of about three times higher in women than in men [12]. Based on histological features and clinical behavior, thyroid cancers are allocated in (a) differentiated thyroid carcinoma (DTC), comprising the papillary (PTC) and follicular (FTC) histotypes; (b) poorly differentiated thyroid carcinoma (PDTC); (c) undifferentiated or anaplastic thyroid carcinoma (ATC) [13]. Total thyroidectomy followed by ^131^I treatment represents the main therapeutic choice for most patients affected by DTC, resulting in a 10-year survival rate of about 90% [14]. On the contrary, a poor prognosis characterizes patients with PDTC and ATC, in which the reduced expression of the natrium/iodide symporter (NIS) renders the ^131^I treatment useless, and external-beam irradiation or chemotherapy is equally ineffective over time [15,16]. In particular, patients affected by ATC have the worst prognosis, with a survival time of a few months from diagnosis. The tumor mass grows rapidly, causing locoregional symptoms, such as dyspnea, dysphagia, and neck pain, and ultimately death occurs following airway obstruction [15,16]. 

The American Thyroid Association (ATA) guidelines recommend the use of cytotoxic radio- and/or chemotherapy as the first-line approach for ATC patients with unresectable or advanced disease [17]. However, these treatments should only be used as interim strategies while the tumor is being characterized and replaced as soon as possible by mutationally specified or molecularly targeted therapies selected according to the tumor features [17,18]. In this view, it is undoubted that the improved knowledge of the molecular mechanisms responsible for ATC progression and drug resistance onset is of utmost importance, as is the testing of drugs potentially capable of interfering with these processes [17,18]. Epidemiological data suggest that aspirin may benefit a broad spectrum of cancer patients. Although it would certainly not be able to eradicate a tumor as monotherapy, aspirin could nevertheless exert therapeutic actions in a combined administration regimen with other drugs. To our knowledge, there is currently no information available on the impact of aspirin treatment on ATC. On this basis, in the present study, we sought to assess the potential anticancer efficacy of ASA on a panel of human ATC-derived cell lines.

## 2. Materials and Methods

### 2.1. Cell Cultures

The human ATC-derived cell lines 8305C, 8505C, and CAL-62 were purchased from DSMZ (Braunschweig, Germany). All these cells have a loss of function mutations of the p53; moreover, 8305C and 8505C cells bear the BRAF^V600E^ mutation, and CAL62 cells harbor a kRAS activating mutation [19]. The 8305C and 8505C cells were cultured in RPMI-1640 and CAL-62 in DMEM. Basal media were supplemented with 10% fetal bovine serum (FBS), 2 mM L-glutamine, and 100 IU/mL Penicillin-Streptomycin at 37 °C in a 5% CO_2_ humidified atmosphere. The acetylsalicylic acid (ASA) was acquired in powder form (Merck Life Science S.r.l., Milan, Italy) and dissolved in culture medium. In all the experiments, media with or without ASA were changed every other day up to the end of the incubation time.

### 2.2. Proliferation Assay

ATC cells were seeded in 96-well plates (2000 cells/well) in quadruplicate. The day after, cells were treated with increasing doses of ASA (from 0.05 to 10 mM) for 72 h. Finally, the tetrazolium salt WST-1 was added (10 μL/100 μL medium/well), and the absorbance was read 4 h later using a microplate ELISA reader (Tecan Group Ltd., Männedorf, Switzerland). The MyCurveFit online tool (https://mycurvefit.com/, accessed on 18 September 2024) was used to create a 4-parameter logistic curve from which IC_50_ values were calculated for each cell line. The time-dependent effects of ASA on ATC cell proliferation were evaluated using ASA 10 mM for CAL62 and 5 mM for 8305C and 8505C cells.

### 2.3. Apoptosis

Cells were seeded in 96-well plates (2500 cells/well) in quadruplicate, and the next day, they were treated with ASA 10 mM for CAL62 and 5 mM for 8305C and 8505C. After 48 h of incubation, cells were lysed and analyzed for the presence of cytoplasmic histone-associated DNA fragments with the Cell Death Detection Elisa^PLUS^ kit (Roche Diagnostics, Monza, Italy), according to the manufacturer’s instructions.

### 2.4. Western Blot

Cells were cultured with or without ASA at IC_50_ concentrations for 24 h and 48 h; then, protein extracts were prepared with RIPA buffer containing fresh-added protease and phosphatase inhibitors. A total of 30 μg of proteins were separated in SDS-PAGE and transferred onto nitrocellulose membranes. Nonspecific binding sites on membranes were blocked by saturation with 5% nonfat dry milk in TBS-Tween buffer for 2 h, then incubated overnight at +4 °C with primary antibodies anti-phospho (Thr308) AKT1/2/3 1:500 (sc-16646, Santa Cruz Biotechnology, Inc., Heidelberg, Germany), anti-AKT1/2/3 1:500 (sc-8312, Santa Cruz Biotechnology), anti-phospho (Thr 202/204) ERK1/2 1:1000 (sc-16982, Santa Cruz Biotechnology), and anti-vinculin 1:10,000 (ab129002, Abcam Inc., Cambridge, MA, USA). After washing, membranes were incubated with anti-rabbit or anti-mouse HRP-conjugated secondary antibodies diluted 1:10,000 (Thermo Fisher Scientific, Rockford, IL, USA). The immunobands were detected using the LiteAblot EXTEND chemiluminescent substrates (Euroclone, Milan, Italy) and the iBright1500 instrument (Thermo Fisher Scientific, Waltham, MA, USA). The relative intensities of signals were quantified by densitometric analysis using the iBright software (version 5.2).

### 2.5. Migration Assay

Migration assays were performed for each cell line with ASA IC_50_ concentrations, as previously described [20]. Briefly, ATC cells were seeded onto 60 mm dishes and grown until 100% confluence was reached. After 3 h of pre-incubation in medium ± ASA, 3 scratches per dish were made in the monolayers with a p200 pipette tip. Dishes were washed and repleted with fresh medium ± ASA, and 10 μg/mL of mitomycin C was added to all cultures to prevent cell proliferation. The scratches were marked at specific points, photographed immediately (T0), and then rephotographed at different time intervals (T1) over a 24-h period with the Moticam 2500 digital camera connected to the microscope Motic-BA410 (Motic, Barcelona, Spain). Areas (A) not covered by cells were measured using the ImageJ software (version 1.48), and the closure speed (S) of the scratches was calculated for each culture as S = (initial A − final A)/T1.

### 2.6. Invasion Assay

At first, Corning^®^ BioCoat control inserts with 8.0 µm pore polyester (PET) membranes (Merck Life Science) were coated with extracellular matrix (ECM) from EHS mouse sarcoma (Merck Life Science) diluted in serum-free culture medium at 0.6 μg/μL and left to solidify at 37 °C overnight. Adherent cells were detached with trypsin/EDTA, centrifuged, resuspended in a serum-free medium, and then seeded onto the upper chambers. The lower chambers were filled with the complete medium as a chemoattractant. For cell treatments, ASA at IC_50_ concentrations was added to all media, and the plate was incubated for 12 h at 37 °C and 5% CO_2_. After that, the medium in the upper chamber was discarded, and non-migrated cells were removed by wiping the membrane with a cotton swab. Cells migrated on the other side were fixed with 100% methanol for 15 min, then washed and stained with a solution of 0.5% Crystal Violet and 20% methanol in H_2_O for 5 min. Finally, the membranes were photographed, and for each image, the color intensity was quantified using the ImageJ software (version 1.48). An image of the cell-free membrane was acquired to eliminate background noise due to the presence of pores, and its color intensity was subtracted from all cell images.

### 2.7. Colony Formation in Soft Agar

The effects of ASA on ATC cell colony formation in a semisolid milieu were tested by a soft agar assay as previously described [21]. In brief, Petri dishes containing ATC cells nestled in soft agar were cultured with or without ASA 10 mM for CAL-62 and 5 mM for 8305C and 8505C. The dishes were photographed after two weeks of incubation. Nine images were acquired for each dish, and colonies were counted by means of the ImageJ software, scoring those with a diameter ≥50 μm.

### 2.8. Statistics

The data are presented as the mean ± standard deviation (SD) or ±standard error (SE) of at least three replicates for each experiment. Comparisons between treated and nontreated samples were analyzed by an unpaired 2-sided Student’s *t*-test with the SPSS software (v.27). 

## 3. Results

### 3.1. Dose- and Time-Dependent Effects of ASA on ATC Cells Proliferation

**Figure 1:**
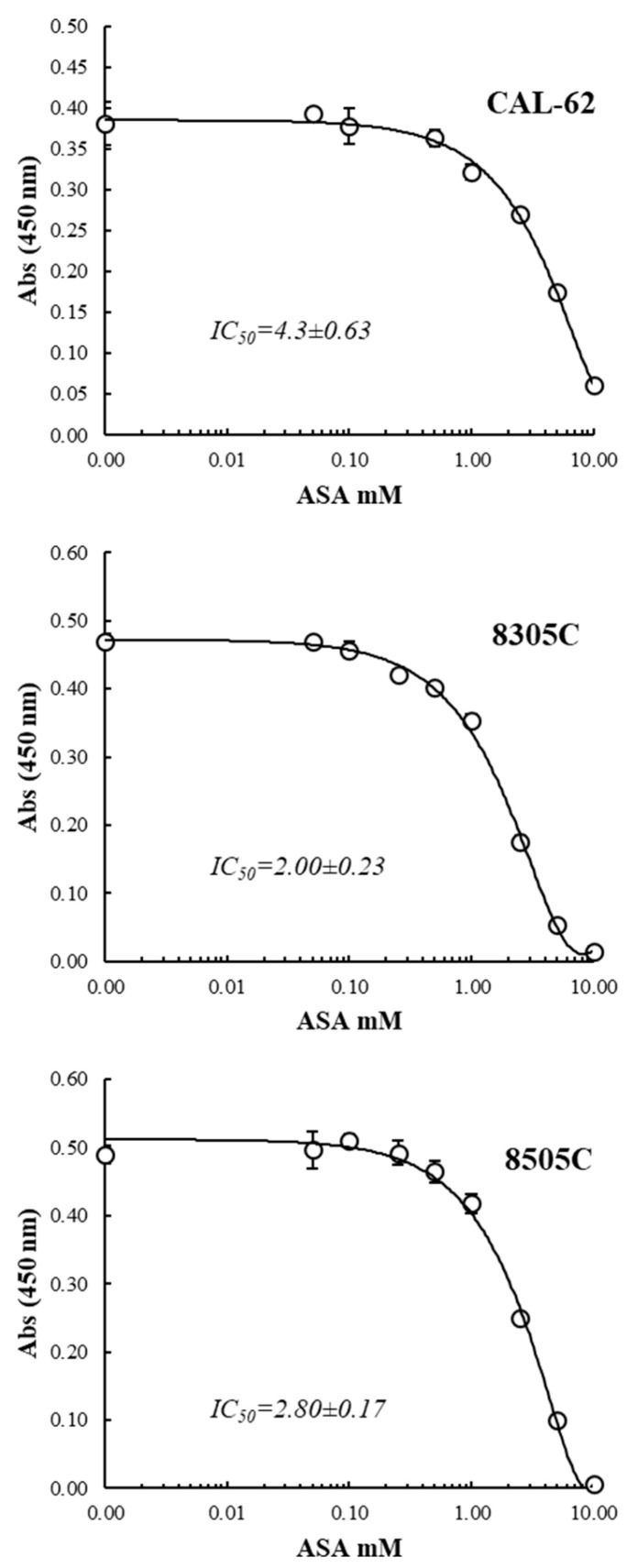
Dose-dependent inhibition of anaplastic thyroid cancer (ATC)-derived cell lines proliferation by ASA. Cells were treated with increasing doses of acetylsalicylic acid (ASA) (from 0.05 to 10 mM) for 72 h. Data are reported as the mean ± standard deviation (SD).

We first evaluated whether ASA could affect the proliferation of the ATC-derived cell lines. As shown in Figure 1, the results obtained clearly demonstrated the inhibition of CAL-62, as well as 8305C and 8505C cell proliferation in a dose-dependent manner with IC_50_ comprised between 2.0 and 4.3 mM.

**Figure 2:**
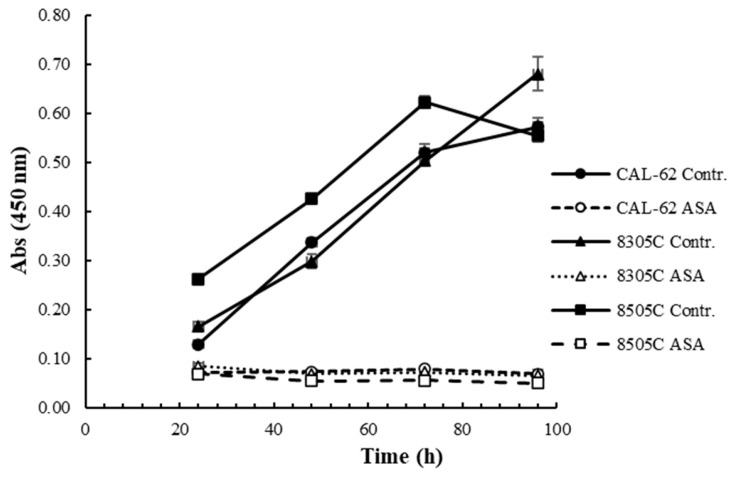
Time-dependent inhibition of ATC-derived cell lines proliferation by ASA. Cells were seeded in 96-well plates, treated with ASA (10 mM for CAL-62, 5 mM for 8305C and 8505C), and measured at 24-h time intervals. Data are reported as the mean ± SD.

Next, we explored the time-dependent effects of ASA on cell proliferation. As evident from Figure 2, cell proliferation was totally and constantly inhibited, confirming the strong antiproliferative effects of ASA over time. 

### 3.2. Apoptotic Effects of ASA on ATC Cells 

**Figure 3:**
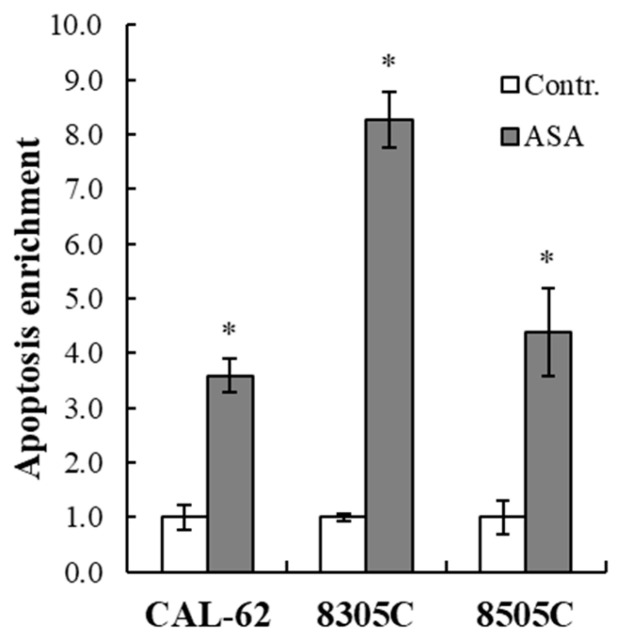
Apoptotic effects of ASA on ATC-derived cell lines. Cells were seeded in 96-well plates and treated with ASA for 48 h. At the end of the incubation time, apoptosis was assessed using the Cell Death Detection ElisaPLUS kit to determine cytoplasmic histone-associated DNA fragments. The enrichment was calculated as the absorbance ratio between treated and non-treated cells. Bars represent the mean ± standard error (SE) of three independent experiments. * *p* < 0.05.

In view of the antiproliferative effects observed, we evaluated whether apoptosis was induced in the ATC cells. To this end, the cell lines were exposed to ASA (10 mM for CAL-62, 5 mM for 8305C and 8505C) for 48 h. The results in Figure 3 demonstrated a significant increase in apoptotic cells in all the cultures following ASA treatment.

### 3.3. ASA Effects on ATC Cells Anchorage-Independent Growth

**Figure 4:**
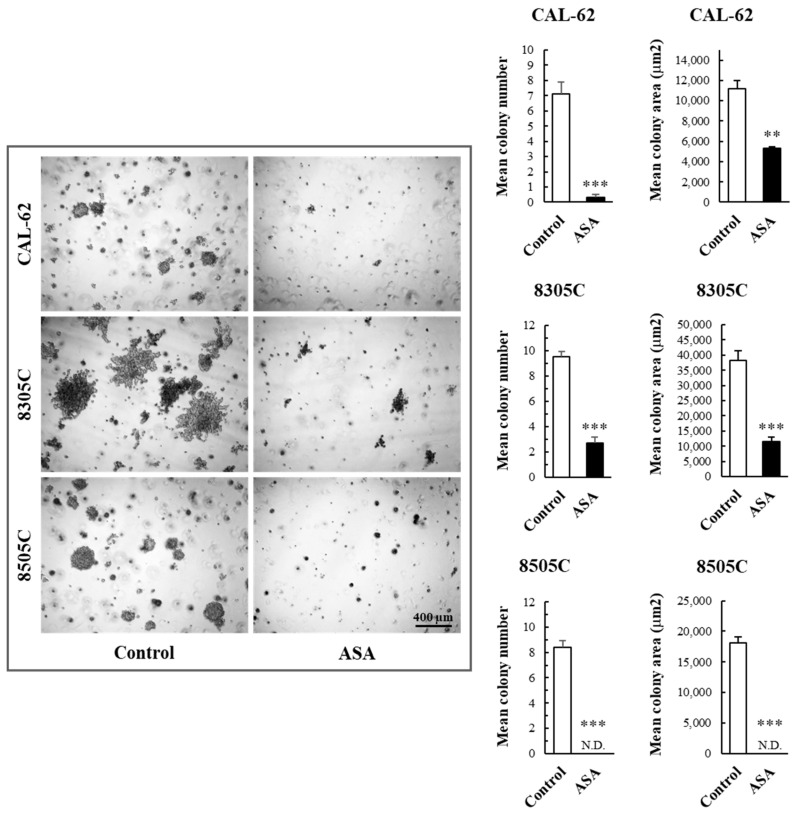
Effects of ASA on the anchorage-independent growth of ATC cells. Cells were grown in a soft agar gel mixed with cell culture medium ± ASA (10 mM for CAL-62, 5 mM for 8305C and 8505C) for two weeks. Photos were finally acquired, and colonies having diameter ≥50 μm were counted. Bars represent the mean ± SE of three independent experiments. N.D., not detectable. ** *p* < 0.01; *** *p* < 0.001.

Next, we examined the ability of ATC cells treated with ASA to grow in an anchorage-independent manner. As shown in Figure 4, exposure to ASA strongly impaired the ability of all the cell lines to form tridimensional colonies in a soft agar layer over time.

### 3.4. ASA Effects on ATC Cells Migration and Invasion

**Figure 5:**
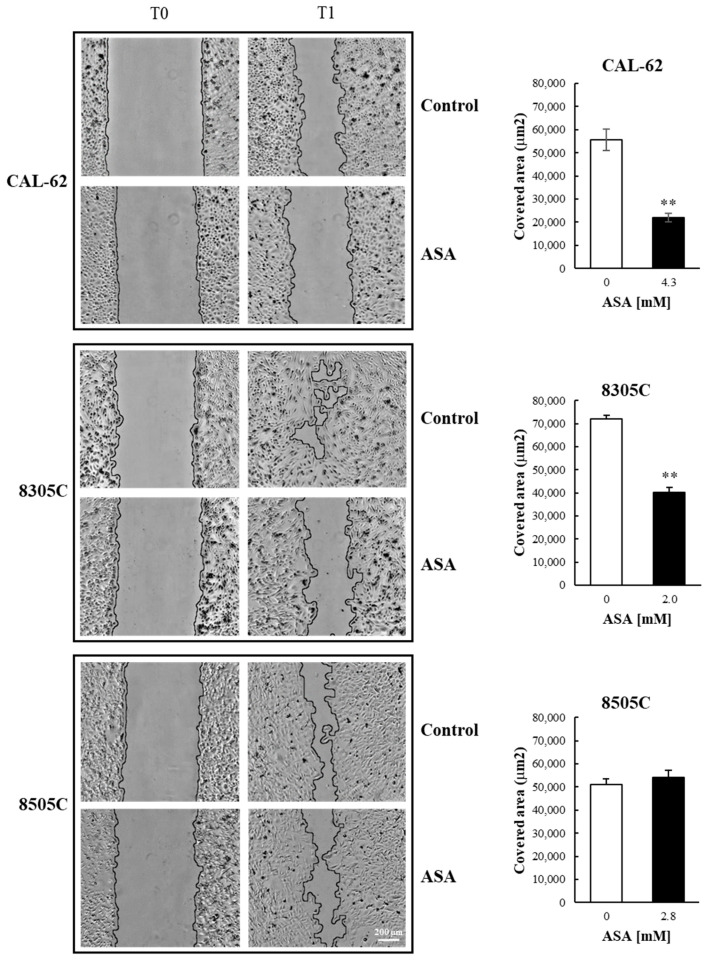
Effects of ASA on ATC cell migration in adherent cultures. Scratch areas were measured with the ImageJ software at different time intervals, and the speed of cell migration was calculated. Bars represent the mean ± SE of three independent experiments. **, *p* < 0.01.

The effects of ASA on the motility of the ATC cells were investigated by means of scratch assays. As reported in Figure 5, the motility of CAL-62 and 8305C cells was significantly impaired by ASA, while that of 8505C did not change.

**Figure 6:**
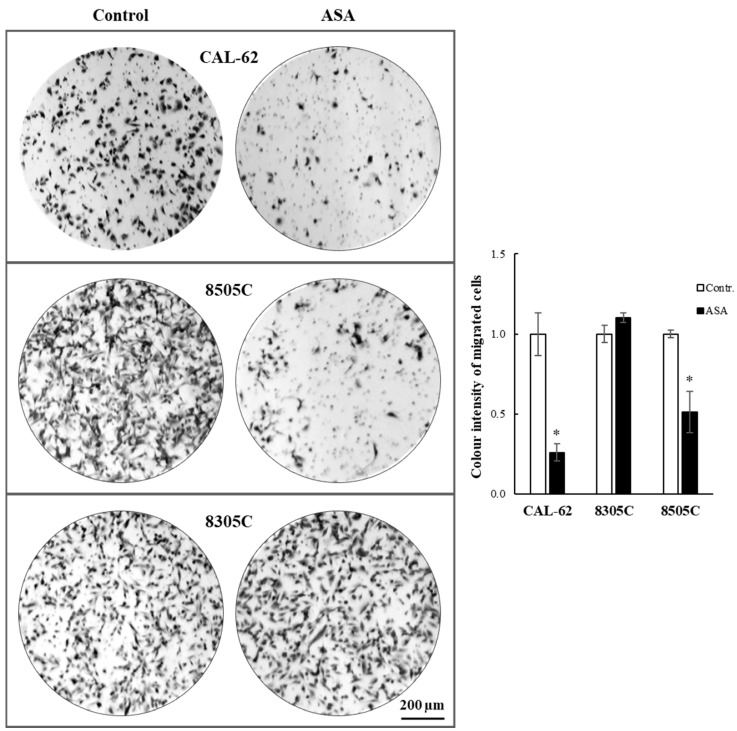
Effects of ASA on ATC cell invasion. Cells were seeded onto PET membranes precoated with ECM in a serum-free medium and incubated for 12 h ± ASA at IC_50_ concentrations. The complete medium was used as a chemoattractant. After removal of non-migrated cells, invading cells were fixed and stained with Crystal Violet. Bars represent the mean ± SE of three independent experiments. *, *p* < 0.05.

Furthermore, the ability of ASA to affect ATC cell penetration through an ECM layer was assessed by invasion assays. Unexpectedly, these results differed partially from those obtained in the previous experiment in that CAL-62 and 8505C displayed a significant reduction of invading cells, while 8305C did not (see Figure 6).

### 3.5. ASA Effects on the MAPK and PI3K/Akt Signaling Pathways in ATC Cells

**Figure 7:**
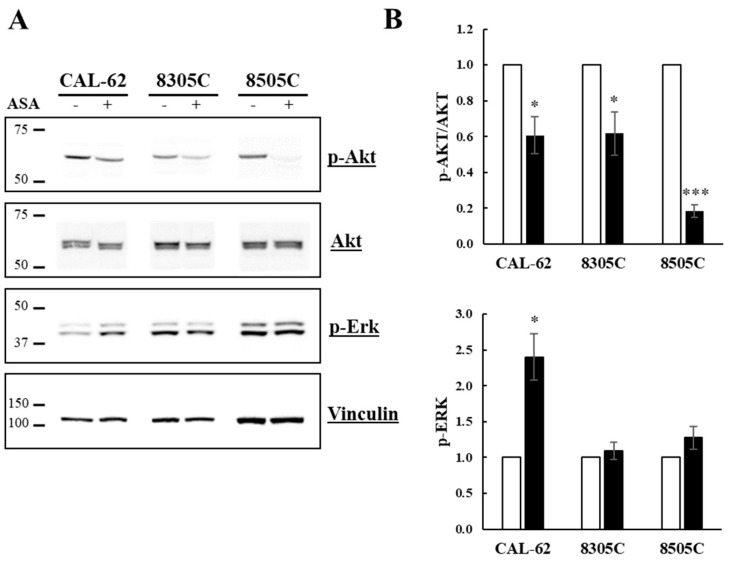
Phosphorylation status of Akt and MAPK in ATC-derived cell lines treated with ASA. Cells were incubated for 24 h ± ASA (10 mM for CAL-62, 5 mM for 8305C and 8505C), then protein extracts were prepared and analyzed by Western blot. Panel (**A**) Images from western blot. Panel (**B**) densitometric analyses. Bars represent the mean ± SE of three independent experiments. *, *p* < 0.05; ***, *p* < 0.001. Original western blots are presented in File S1.

We finally investigated the effects of ASA on the MAPK and PI3K/Akt signaling pathways, which are often found altered in ATC cells [22]. As reported in Figure 7 (panel A), the phosphorylation status of Akt1/2/3 kinases was significantly reduced following 24 h of treatment with ASA. On the other hand, ERK1/2 phosphorylation was either unaffected (8505C) or slightly upregulated (CAL-62 and 8305C).

## 4. Discussion

Despite the considerable increase in our knowledge of the molecular pathogenesis of ATC, effective therapies to ameliorate the very poor prognosis of this deadly disease are not yet available [15,16,17,18,22]. Over the last five decades, experimental and clinical evidence indicated a potential therapeutic value of ASA in different human cancers, especially colorectal, breast, and prostate neoplasms [1,2,3,4,5,6,7,8,9,10,11,12]. In addition, a number of other solid and liquid tumors may also benefit from the therapeutic effects of ASA [10,11]. Overall, observational studies evidenced that the use of aspirin is associated with a 20–30% reduction in cancer incidence and mortality [11]. It has to be mentioned, however, that a large randomized clinical trial (ASPREE, Aspirin in Reducing Event in the Elderly) in which more than 19,000 participants were enrolled reported higher all-cause mortality among individuals receiving daily low-dose aspirin compared to those who took placebo, with 1.6 excess deaths per 1000 person-years occurring in the aspirin group after 4.7 years, for which cancer was the main cause [11,23,24,25]. A Korean nationwide nested case-control study specifically focused on thyroid tumors found no difference in thyroid cancer development between aspirin users and non-users over a 14-year observation period. In this study, the median daily dose of aspirin for patients treated for more than 30 days per year was 100 mg. Therefore, based on these findings, it could be argued that the low dose of aspirin is insufficient to exert a preventive effect against cancer [26]. Recently, a 20-year cohort study comprising more than one million cancer-free individuals at baseline compared the incidence of first cancer among new users of low-dose (75–150 mg) or high-dose (500 mg) aspirin and non-users [27].

This work evidenced that low-dose aspirin did not reduce the hazard ratio (HR) for cancer overall, irrespective of continuity and duration of use. However, contrary to the above studies, prolonged consumption for more than 5 or 10 years was found to be associated with at least a 10% reduction in HR for several cancer types, including thyroid carcinomas (HR~0.7). In addition, consistent high-dose aspirin use was associated with HR reduction by 10% or more in all cancers and halved in thyroid tumors (HR = 0.48) [27].

These conflicting observations could depend on several reasons, such as classification criteria of aspirin users, sample size, statistical methods, confounding variables, and limitations inherent to observational studies. In this context, in vitro experiments could give a relevant contribution by producing direct evidence of anticancer actions to support epidemiological data. To our knowledge, only one study has explored the effect of aspirin, alone and combined with metformin, on the papillary thyroid cancer cell line TPC-1. The authors documented that aspirin could reduce cell proliferation and induce apoptosis, and these effects were enhanced by combining aspirin and metformin [28].

To the best of our knowledge, our study represents the first preclinical investigation of the in vitro effects of ASA on a panel of three anaplastic cancer-derived cell lines. The results obtained demonstrated the ability of ASA to inhibit proliferation and trigger apoptosis of all the ATC cell lines in a time- and dose-dependent manner, with IC_50_ in the lower millimolar range (2–4 mM). These concentrations are similar to those employed on TPC-1 and in previous studies evaluating the effects of ASA on the proliferation of different cell types [28,29,30,31]. In addition, ASA inhibited cell migration and invasion of at least two ATC cell lines while drastically reducing the anchorage-independent cell growth in a semisolid milieu of all three ATC lines. 

The PI3K/Akt and MAPK mitogenic pathways are boosted during thyroid cancer progression, prompting cell proliferation and survival [22,32]. Here, we report the ability of ASA to induce a strong decrease of Akt phosphorylation in all the ATC cell lines evaluated, while it shows little or no effects on the phosphorylation status of ERK 1/2. It may be assumed that interference with the Akt signal most likely contributes to the antiproliferative action observed in our experimental setting. However, more extensive studies are needed to fully explore the potential effects of ASA on other stimuli involved in ATC cell growth. In particular, ASA was also shown to inhibit the NF-κB and Wnt signaling paths, both involved in the progression of aggressive thyroid cancers [2,13,22,30,31,32,33,34,35]. 

In considering the possible clinical translation of the present data, it has to be mentioned that ASA effects on ATC cell proliferation, survival, and in vitro tumorigenicity were obtained with doses in the lower millimolar range, higher than plasma concentrations, in the micromolar range, observed following low-dose (100 mg) aspirin treatment [36], and those required to affect COX-2 activity in different cell types [37]. However, it is worth considering that therapeutic concentrations of ASA reach up to 1.67 mM (30 mg/dL), which is very close to the antiproliferative IC_50_ values observed in our experimental setting and consistent with the epidemiological observation that the use of high-dose (500 mg) aspirin is associated with reduced thyroid tumor HR [27,38]. 

## 5. Conclusions

In the present manuscript, we report evidence showing the ability of aspirin to inhibit ATC cell proliferation, survival, and in vitro tumorigenicity, which warrants further investigations to assess its therapeutic usefulness in patients affected by ATC, still characterized by a dismal prognosis. 

## Data Availability

The data presented in this study are available on request from the corresponding author.

## Supplementary Materials

The following supporting information can be downloaded at: https://www.mdpi.com/article/10.3390/cancers16244203/s1, File S1: Original western blots.
