# Supplementary figures and images for: The Potential Therapeutic Value of Aspirin in Anaplastic Thyroid Cancer
Source: Cancers (Basel). 2024 Dec 17;16(24):4203. doi: 10.3390/cancers16244203 (PMC11674608; doi:10.3390/cancers16244203)

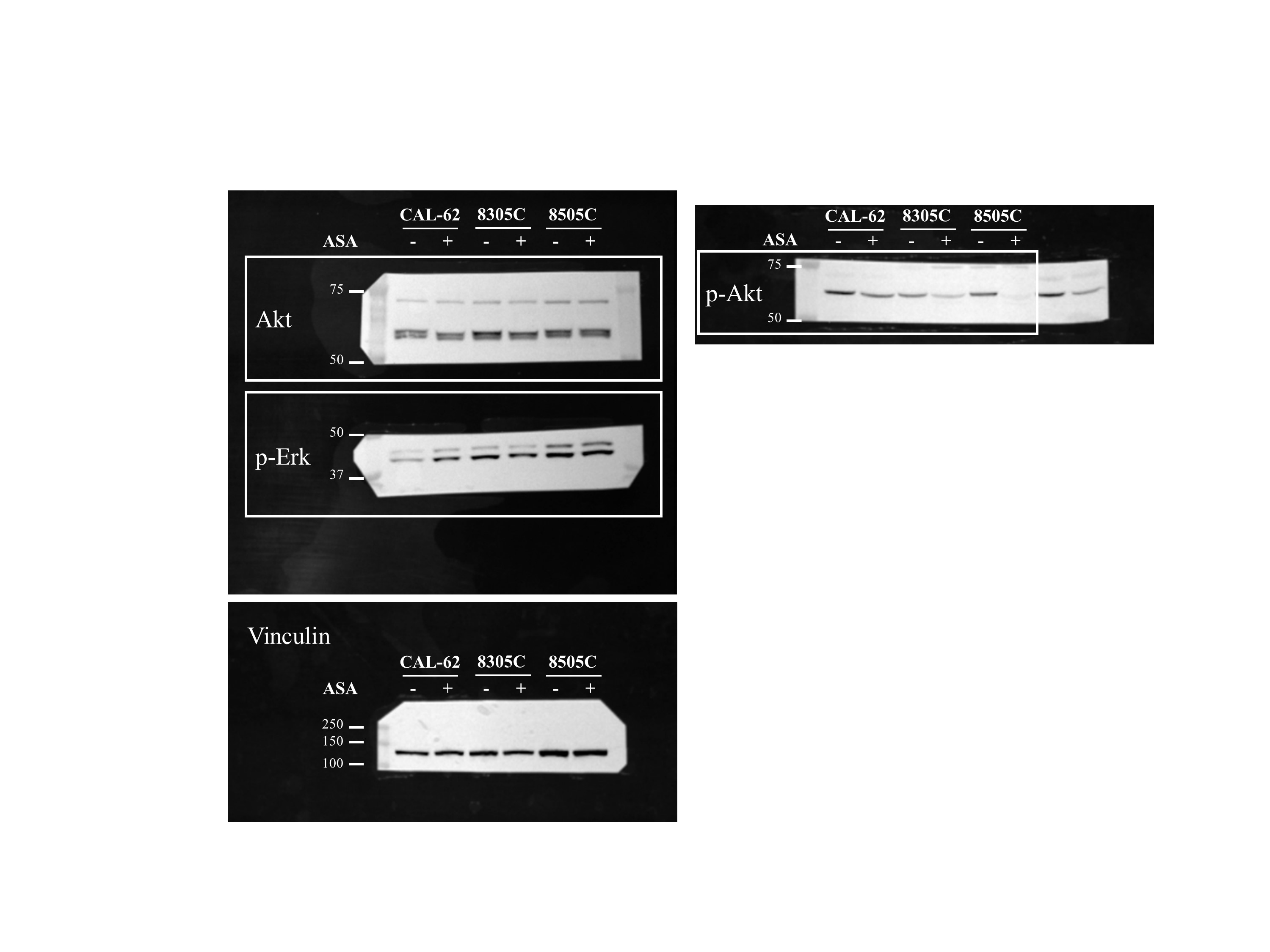

Supplement: Supplementary file 1 [file cancers-16-04203-s001.zip › cancers-3326363-supplementary.jpg]
